# Effectiveness of Various Fluoride Varnishes in Preventing Dental Caries in Children and Adolescents: A Systematic Review of Evidence From Educational Settings

**DOI:** 10.7759/cureus.96323

**Published:** 2025-11-07

**Authors:** Amjad S Almalki, Omar S Alshahrani, Fetoon A Aljoaid, Melaf A Alrwiali, Rnda A Alrais, Alanoud B Alfuhigi, Raghad A Alfehaid, Reham M Daghriri, Abdulmajeed N Alwably, Ghadah M Alenazi, Ahmed A Alasmari

**Affiliations:** 1 Pediatric Dentistry, Dental Center, Prince Sultan Military Medical City, Riyadh, SAU; 2 Pediatric Dentistry, Armed Forces Hospital Southern Region, Khamis Mushait, SAU; 3 Pediatric Dentistry, Al-Baha University, Al-Baha, SAU; 4 College of Dentistry, Al Jouf University, Sakakah, SAU; 5 General Dentistry, King Saud University, Riyadh, SAU; 6 General Dentistry, Jazan University, Jazan, SAU; 7 College of Dentistry, Qassim University, Buraydah, SAU; 8 Pediatric Dentistry, Riyadh Second Health Cluster, Health Holding Company, Riyadh, SAU; 9 Orthodontics and Dentofacial Orthopaedics, Armed Forces Hospital Southern Region, Khamis Mushait, SAU

**Keywords:** children’s dental care, oral health prevention, preventive interventions, public health dentistry, school based fluoride delivery

## Abstract

Dental caries is a chronic oral disease that affects children and adolescents worldwide, significantly impacting oral health and quality of life. Fluoride varnish (FV) application is an effective preventive intervention to reduce the incidence and increment of caries, particularly when delivered within educational settings. This systematic review collates evidence from 14 randomized controlled trials (RCTs) conducted across diverse geographic and socioeconomic contexts. The primary objective is to assess the efficacy of various fluoride interventions delivered in educational settings. PubMed, Cochrane Controlled Register of Trials (CENTRAL), and Google Scholar databases were searched for studies that met the inclusion criteria of focusing on FV application in children in educational settings with a minimum six-month follow-up. The Risk of Bias 2 (RoB 2; Cochrane, London, United Kingdom) tool was used to evaluate methodological quality, and Review Manager (RevMan), Version 7.12.0 (2024; Cochrane) was used for quantitative synthesis. The combined data from more than 13,000 children who received FV showed a consistently lower risk of dental caries than that of the controls, with risk ratios strongly supporting the intervention. School-based FV delivery systems are feasible, and their economic value appears to vary depending on program design and local resources. The preventive impact of FV was strong, despite differences in study designs, caries evaluation techniques, and follow-up times. However, to maximize policy implementation, long-term assessments using standardized outcomes such as child-level caries incidence, DMFT (Decayed, Missing, and Filled Teeth) increments, and Dental Fear Survey (DFS) scores reported at common time points are needed. In summary, this review offers compelling data supporting the use of FV as a safe and successful method for preventing dental cavities in children and adolescents in educational settings. The results support the widespread inclusion of FV in school-based health services worldwide and reaffirm its importance as a component of comprehensive oral health promotion programs.

## Introduction and background

Dental caries, a multi-factorial chronic oral disease that affects most populations, is considered the most important oral health burden globally [1]. It can begin in early childhood and progress throughout life, despite being preventable [2]. Early childhood caries (ECC) is characterized by carious lesions that invade the dental hard tissue on deciduous tooth surfaces, and it occurs within the first three years of life in infants and toddlers [3]. In permanent teeth, carious lesion development is most likely to occur in the first few years after tooth eruption [1]. Untreated caries causes progressive destruction of the crowns of the teeth, often accompanied by severe pain and suffering [4]. This can result in failure to thrive and poor school performance, which can impact a child’s short- and long-term future [2]. 

Dental caries affects approximately 40% of children aged seven and reaches 85% prevalence by the age of 17 years [5]. In the United States alone, nearly 40% of young children experienced caries between 2015 and 2016, underscoring the substantial public health burden of ECC. Worldwide, the direct costs of treatment for dental caries were estimated to be approximately $298 billion yearly in 2010 [6]. The financial burden also affects families via missed work, childcare, and school days, as well as society in terms of lost productivity from the time spent sending children to receive complicated dental care for a preventable issue. 

Given this burden, preventive strategies should be a public health priority because they can significantly reduce the formation and progression of dental caries. Since the discovery of fluoride in the 1940s, public health systems and the oral health product industry have made important advances in the control of oral diseases [7]. Topical application of fluoride gel and supplements is a convenient and inexpensive method for reducing dental caries [8-11]. In 2014, Marinho et al. reported a series of Cochrane reviews discussing the available evidence on the ability of fluoride therapies to prevent dental caries. The major findings from the reviews were that for topically applied fluoride treatments, there were clear decreases in caries increment in permanent and primary dentitions for all forms of therapies and fluoride varnishes [9]. There is compelling evidence that fluoride varnish (FV) prevents caries, with approximately 37% of caries reduction in primary dentition and 47% in permanent dentition [4]. Additionally, FVs are easy to apply and well tolerated, and FV application requires only one to four minutes per patient [10]. 

Due to increasing inequities in dentistry, together with rising healthcare costs and resource restrictions, there is a need for tailored, risk-based preventative and therapeutic approaches that provide high value in return for the interventions. The United States Preventive Services Task Force (USPSTF) makes recommendations for preventive services delivered in primary care settings, including FV treatments for children from the time the first tooth erupts to five years of age, but found insufficient evidence for youth aged 5-17 years [8]. This proves that schools are an important setting for the delivery of FV. In 2022, approximately 70% of state oral health programs reported having at least one school-based FV delivery program (SFVDP) [8]. Australia’s National Oral Health Plan also recognizes that the application of FV is a safe, effective, and efficient strategy for promoting oral health and reducing dental decay in vulnerable populations [11].

This systematic review aims to consolidate the current evidence on the effectiveness of school-based fluoride delivery programs in preventing dental caries in children and adolescents. It builds on prior systematic reviews by specifically synthesizing evidence from educational settings, thereby addressing the gap left by earlier meta-analyses that did not isolate this context. The primary objective is to systematically evaluate randomized controlled trials (RCTs) to assess the efficacy of various fluoride interventions delivered in educational settings across different developmental stages, including early childhood to adolescence. The secondary objective is to analyze the cost-effectiveness of fluoride interventions and their impact on addressing health disparities. By ensuring that all included data meet the highest scientific standards, this review is able to inform clinical practice and public health policy for future research on school-based fluoride delivery programs. 

## Review

Methods

The review methodology was designed to assess the efficacy of different fluoride varnish formulations and application techniques in reducing dental caries in children and adolescents in school-based settings. The Preferred Reporting Items for Systematic Reviews and Meta-Analyses (PRISMA) 2020 guidelines were used to ensure procedural transparency and quality. 

Eligibility Criteria

Inclusion Criteria: The Population, Intervention, Comparison, Outcomes, and Study (PICOS) framework was used to outline the research protocols. The following inclusion criteria were used: (i) Population: Children enrolled in educational settings (preschools, nursery schools, Head Start programs, primary schools, and middle schools), (ii) Intervention: Professionally applied FV, at any frequency (quarterly, semi-annual, or annual) within educational settings with a minimum six-month follow-up, (iii) Comparison: Groups included no treatment, placebo varnish, standard care, or alternative fluoride interventions, (iv) Outcomes: Caries incidence or caries increment as primary endpoints, with cost-effectiveness as a secondary outcome, and (v) Studies: RCTs (individual or cluster), studies published in peer-reviewed journals (with adequate sample sizes for statistical analysis) and with documented parental or guardian consent. 

Exclusion criteria: Methodological, setting-based, and publication exclusions were observational studies, quasi-experimental studies, systematic reviews and meta-analyses, and qualitative studies, investigations with follow-up periods shorter than six months, studies lacking appropriate control groups or those with significant design flaws, studies lacking appropriate ethical approval, studies conducted exclusively in clinical or home-based environments, and conference abstracts, protocol papers, and dissertations. No geographic or time restrictions were applied to capture evidence across diverse populations and implementation contexts. Non-English publications were excluded.

Database Search Strategy 

A comprehensive literature search was conducted across three databases: PubMed Central/MEDLINE (Medical Literature Analysis and Retrieval System Online), Cochrane Central Register of Controlled Trials (CENTRAL), and Google Scholar. Search strategies were adapted appropriately for each database while maintaining consistent terminology and scope. The search strategy combined Medical Subject Headings (MeSH) and keywords using Boolean operators. The detailed search strategies tailored to each database, outlining the specific combinations of keywords, MeSH terms, and Boolean operators used, are summarized in Table 1. Searches covered the entire span of database records from inception through September 2025 and included publications in English and articles with English abstracts. The search was further refined by limiting the retrieval to RCTs.

**Table 1:** Database-specific search strategies used in the review

Database	Search Strings
PubMed Central/MEDLINE	("fluorides"[MeSH Terms] OR "varnish"[All Fields] OR "varnishes"[All Fields]) AND (("caries"[All Fields] OR "dental caries"[MeSH Terms] OR "dental caries"[All Fields] OR "caries"[All Fields]) AND "prevention and control"[MeSH Subheading] OR )) AND ("child"[MeSH Terms] OR "children"[All Fields])
Cochrane Central Register of Controlled Trials (CENTRAL)	(fluoride varnish OR topical fluoride) AND (dental caries OR caries prevention) AND (school OR kindergarten OR preschool) AND (child OR children OR pediatric)
Google Scholar	"fluoride varnish" "school-based" "caries prevention" "dental caries" "children"

Screening and Study Selection

Since Google Scholar indexing can vary, all records retrieved from Google Scholar were cross-checked against PubMed and CENTRAL to identify and remove duplicates. Titles and abstracts from Google Scholar were screened using the predefined eligibility criteria to ensure quality and consistency, and only peer-reviewed journal articles meeting the inclusion criteria were retained. Initially, all retrieved citations underwent title and abstract screening using the predefined eligibility criteria. Full-text articles were then acquired for studies that fulfilled the initial screening criteria and were assessed for final inclusion.

Data extraction was performed using a predetermined, standardized form. Two independent reviewers extracted data elements, such as study characteristics, participant demographics (including age range, sample size, baseline caries status, and socioeconomic indicators), and detailed intervention attributes (such as FV type and concentration, application frequency, technique, and operator qualifications).

Risk of Bias Assessment 

Risk of bias assessment was performed using the Cochrane risk-of-bias tool (ROB 2), which is a standardized framework comprising five domains that assess the potential for bias in randomized controlled trials [12]. The five predefined domains were as follows: (i) bias arising from the randomization process, (ii) bias due to deviations from the intended interventions, (iii) bias resulting from missing outcome data, (iv) bias in outcome measurement, and (v) bias in the selection of the reported data. Two independent reviewers assessed each of the included trials, and any disagreements were resolved by discussion. The RCTs were classified into three categories: “low risk,” “high risk,” and “some concerns” based on the inherent limitations across the evaluated domains. 

*Statistical Analysis* 

Review Manager (RevMan), Version 7.12.0 (2024; Cochrane, London, United Kingdom) was used to quantitatively assess the studies. A forest plot was used to visualize the meta-analysis results. The random effects (RE) model was chosen because of the variability in FV formulations, application regimens, participant demographics, and outcome measurement methods in the included studies. Heterogeneity was quantified using the I² statistic, with values above 50% considered substantial. In cases where meta-analysis was not feasible, a narrative synthesis was provided. To assess the publication bias, a funnel plot was generated, and to complement the synthesis of the outcomes, the overall certainty of evidence was evaluated using the GRADE (Grading of Recommendations Assessment, Development and Evaluation) approach. 

Results 

Study Selection

**Figure 1:**
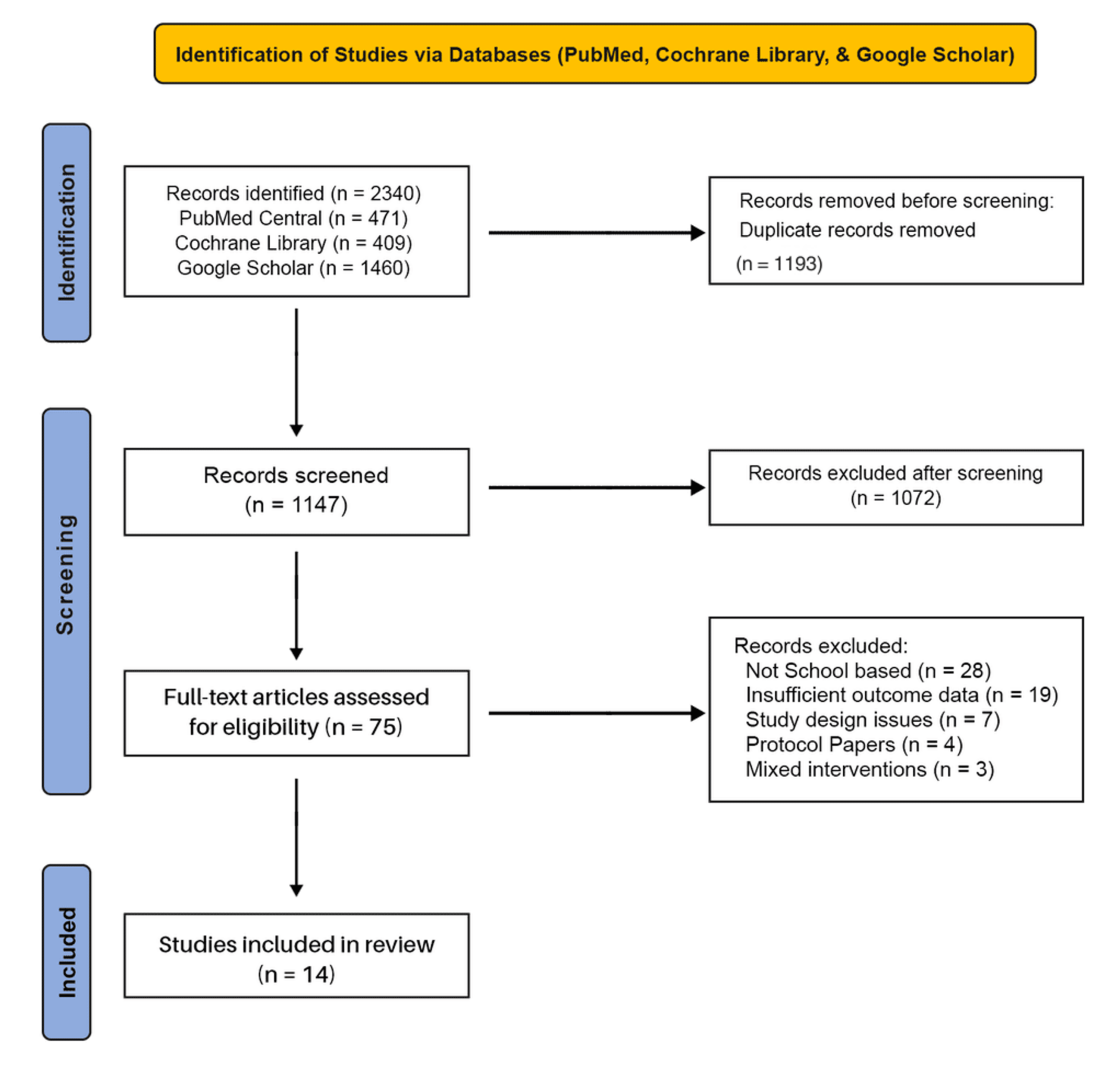
PRISMA 2020 flow diagram summarizing the database search and article selection process PRISMA: Preferred Reporting Items for Systematic Reviews and Meta-Analyses

The screening process identified 2340 articles in PubMed Central/MEDLINE, CENTRAL, and Google Scholar. After removing duplicate records, 1147 unique relevant citations remained. Of these, 1072 were excluded primarily due to methodological issues, study design (not school-based), and insufficient outcome data. The remaining 75 full-text articles were screened, and 14 high-quality studies that met all the inclusion criteria were incorporated into the final systematic review. Of the 14 included trials, seven were not eligible for quantitative synthesis due to inconsistent outcome measures, heterogeneous follow-up periods, or insufficient statistical reporting. These studies were instead included in the narrative synthesis to ensure comprehensive coverage, as outlined in the PRISMA flow diagram (Figure 1). 

Study Characteristics

In this systematic review, a methodological decision was made to include only RCTs in order to focus on the clinical utility of the evidence. Furthermore, the elimination of selection bias through true randomization and the standardization of evidence quality inherent to RCT designs offer great insights into the establishment of causal relationships, leading to enhanced precision of effect estimates. 

The sample sizes ranged from 60 to 5,397, with all studies relevant to the primary analyses of the effect of FV on dental caries prevention in school-based settings. In addition, the combined dataset comprised over 13,000 children assessed over periods of up to 36 months (Table 2). The characteristics of the included studies are given in Table 3.

**Table 2:** Overall study characteristics.

Characteristic	Value
Total Studies	14
Total Participants	13,263
Median Sample Size	481
Range of Follow-up	12-36 months
Most Common Follow-up	24 months (5 studies)

**Table 3:** Characteristics and outcomes of included studies. ICDAS, International Caries Detection and Assessment System; OHP, oral health promotion; INT, intervention group; UC, usual care group; FPM, first permanent molars; ANOVA, analysis of variance; FV, fluoride varnish; CO, control group; ECC, early childhood caries; dmft, decayed, missing, and filled teeth (primary dentition); d3mft/d3t, decayed, missing, and filled teeth at cavitation threshold; DFS, decayed and filled surfaces; DMFT, decayed, missing, and filled teeth; DMFS, decayed, missing, and filled surfaces.

Study	Objectives	Sample Size	Timing	Follow-up Periods	Key Findings
Agarwal et al. (2022) [13]	To evaluate the effectiveness of fluoride varnish in preventing early childhood caries in high-risk preschool children.	515	36 months	Baseline, 6, 12, 36 months	The baseline ICDAS score increased by 0.182, and the varnish group had a 0.154 lower increment than that of the control.
Braun et al. (2016) [14]	The effectiveness of community-based oral health promotion (OHP) activities in reducing caries among children in American Indian Head Start centers.	1,040	36 months	Baseline, 12, 24, 36 months	The mean oral health behavior scores increased significantly in the INT group compared to the UC group (P = 0.006).
Chestnutt et al. (2017) [15]	To compare the effectiveness of fluoride varnish and fissure seal in preventing dental caries in first permanent molars (FPM).	1,016	36 months	Baseline, 6, 12, 18, 24, 30, 36 months	No significant differences were observed between the interventions. Both were equally effective in preventing caries in the first permanent molars.
de Souza et al. (2021) [16]	This study investigated the acceptance and effectiveness of fluoride varnishes in children.	60	18 months	Baseline, 3, 6, 9, 12, 15, 18 months	Regression of lesions was observed in 13% of the children. No differences in the ICDAS score distribution were found among the groups at baseline and at the end of the study (ANOVA; p > 0.05).
Effenberger et al. (2021) [17]	To assess the effectiveness of fluoride varnish in preventing dental caries in high-risk South African children.	513	24 months	Baseline, 12, 21, 24 months	There were no significant differences in the increment of teeth with cavitated caries lesions or extractions between the FV and CO groups (p > .05).
Jayasinghe et al. (2021) [18]	To evaluate the effectiveness of 6-monthly fluoride varnish application in preventing dental caries in Sri Lankan school children.	321	24 months	Baseline, 6, 12, 18, 24 months	Significant reduction in new caries development at all time points. The mean new caries in the intervention and control groups was 1.50 and 1.97, respectively (p<0.001).
Jiang et al. (2014) [19]	To evaluate the effectiveness of hands-on training in parental toothbrushing, with or without fluoride varnish applications, in preventing early childhood caries (ECC) among young children in Hong Kong.	450	24 months	Baseline, 6, 12, 18, 24 months	The overall mean dmft score at baseline was 0.03±0.24, with no significant differences in dmft scores among the three groups at baseline and 24-month follow-up.
Lam et al. (2021) [20]	To compare the effectiveness of glass ionomer sealants versus fluoride varnish in preventing occlusal caries in primary second molars.	333	12 months	Baseline, 6, 12 months	No significant difference was observed between the interventions (p=0.913). Both were equally effective despite low sealant retention (13.5%).
McMahon et al. (2020) [21]	To determine the effectiveness of fluoride varnish application in nursery school children in Scotland.	1,284	24 months	Baseline, 6, 12, 18, 24 months	Although the primary outcome of the study did not reach statistical significance for the overall worsening of d3mft, there was a statistically significant (p = 0.043) reduction in the worsening of decayed primary teeth (d3t) in the fluoride varnish group.
Muñoz-Millán et al. (2017) [22]	To assess the effectiveness of biannual fluoride varnish applications in preventing early childhood caries in rural Chilean preschool children.	275	24 months	Baseline, 6, 12, 18, 24 months	The caries incidence was 45.0% in the experimental group and 55.6% in the control group after 24 months, with a prevention fraction of 18.9% (P = .081). Reduced dmft increment, but high dropout rates (31%) were observed.
Pisarnturakit and Detsomboonrat (2020) [23]	Comparing intensive versus basic preventive regimens in Thai Kindergarten children.	121	24 months	Baseline, 6, 12, 24 months	No significant additional benefit was observed for intensive protection over basic care (p=0.709). Both approaches were equally effective.
Sirivichayakul et al. (2023) [24]	To evaluate the effectiveness of two fluoride varnishes versus a placebo control on approximal carious lesions in preschool children.	190	18 months	Baseline, 6, 12, 18 months	Fluoride varnish significantly slowed the progression of approximal caries compared to placebo (p<0.001).
Wang et al. (2021) [25]	Assess the effectiveness of fluoride varnish on newly erupted permanent molars in Chinese children.	5,397	36 months	Baseline, 24, 36 months	The mean DFS scores at 24 months were 0.41 (test group) and 0.64 (control group), showing a significant difference (P<0.001).
Wu et al. (2020) [26]	To evaluate the long-term effectiveness of fluoride varnish application over three years in rural Chinese children.	1,748	36 months	Baseline, 6, 12, 18, 24, 30, 36 months	Lower DMFT index and a lower DMFS index in the experimental group compared with the control group (respectively: 58.9% vs. 65.5%, 34.8% vs. 42.1%, 1.38 vs. 1.59, and 2.06 vs. 2.38).

The samples span six continents and a wide range of healthcare systems (Table 4), including rural Asia, national programs in Europe, and multicenter trials in South America and Africa. This variation illustrates the transferability of high methodological standards in FV applications in school-based settings.

**Table 4:** Geographic distribution of the randomized control trials.

Region	Number of Studies	Studies
Asia	8	Agarwal et al. (India) [13], Jiang et al. (Hong Kong) [19], Jayasinghe et al. (Sri Lanka) [18], Lam et al. (Hong Kong) [20], Pisarnturakit et al. (Thailand) [23], Sirivichayakul et al. (Thailand) [24], Wang et al. [25] (China), Wu et al. (China) [26]
Europe	2	Chestnutt et al. (UK-Wales) [15], McMahon et al. (UK-Scotland) [21]
Americas	3	Braun et al. (USA) [14], De Souza et al. (Brazil) [16], Muñoz-Millán et al. (Chile) [22]
Africa	1	Effenberger et al. (South Africa) [17]

Risk of bias assessment: analysis of the quality and reliability of the RCTs 

**Figure 2:**
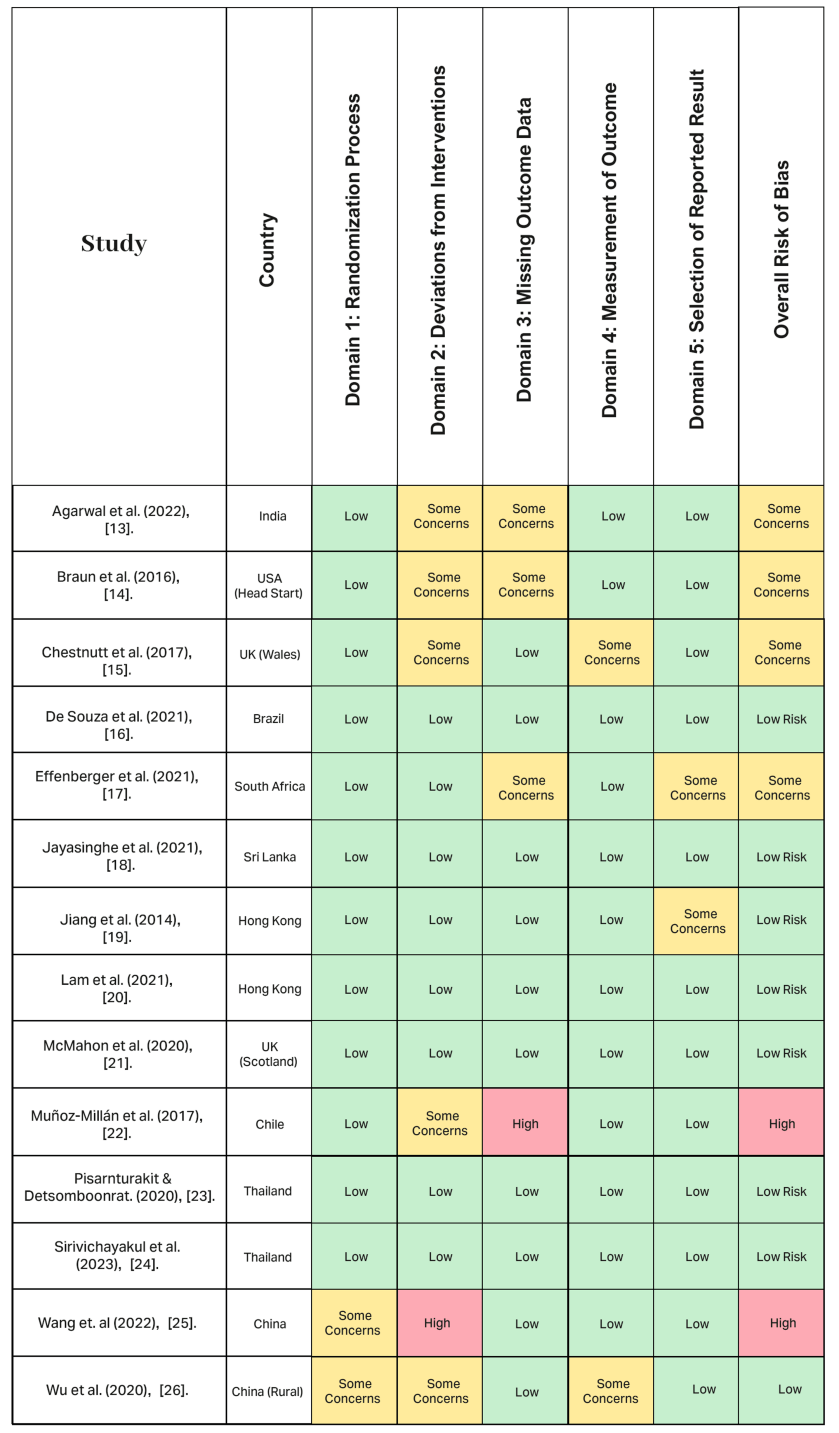
Risk of bias assessment of included randomized controlled trials across five domains Low = Indicates low risk of bias; Some Concerns = Indicates moderate or uncertain risk of bias; High = Indicates high risk of bias Studies included: [13-26]

ROB 2 was used to evaluate the efficacy of school-based fluoride delivery programs in 14 RCTs. The patterns in the assessment (Figure 2) illustrate the intricate balance between scientific accuracy and practical constraints that define the real-world implementation of preventative oral health research. 

The analysis revealed a quality evidence base: only 14.29% (n=2) of studies were labeled “high-risk,” with 28.57% (n=4) of studies labeled as having “some concerns.” The majority of the studies, 57.14% (n=8), had overall “low-risk” classifications (Table 5), providing reliable evidence for this systematic review. 

**Table 5:** ROB 2 assessment summary of included studies

Risk of Bias Level	Number of Studies	Percentage	Studies
Low Risk	8	57.14%	De Souza et al. (2021) [16], Jayasinghe et al. (2021) [18], Jiang et al. (2014) [19], Lam et al. (2021) [20], McMahon et al. (2020) [21], Pisarnturakit et al. (2020) [23], Sirvichayakul et al. (2023) [24], Wu et al. (2020) [26]
Some Concerns	4	28.57%	Agarwal et al. (2022) [13], Braun et al. (2016) [14], Chestnutt et al. (2017) [15], Effenberger et al. (2021) [17]
High Risk	2	14.29%	Muñoz-Millán et al. (2017) [22], Wang et al. (2021) [25]
Total	14	100%	All studies

The evidence suggests competent trial designs, adherence to comprehensive reporting standards, and the use of accurate analytical methods. All trial protocols were prospectively registered in clinical trial registries, either regionally or with ClinicalTrials.gov (at the National Institutes of Health (NIH)), demonstrating adherence to transparent research practices. This has resulted in a body of literature that can form the foundation for evidence-based practice and policy development related to school-based fluoride delivery programs. 

Meta-Analysis 

Of the 14 included randomized controlled trials, eight studies provided complete dichotomous outcome data (number of events and total participants per intervention and control arms) suitable for pooling in a risk ratio (RR) meta-analysis. The data extracted from eight RCTs [15,19,21-26] included in the quantitative synthesis, comprising 11,130 participants (5,407 intervention, 5,723 control) provided adequate comparable data on caries incidence outcomes suitable for meta-analysis pooling. 

Forest Plot Presentation 

**Figure 3:**
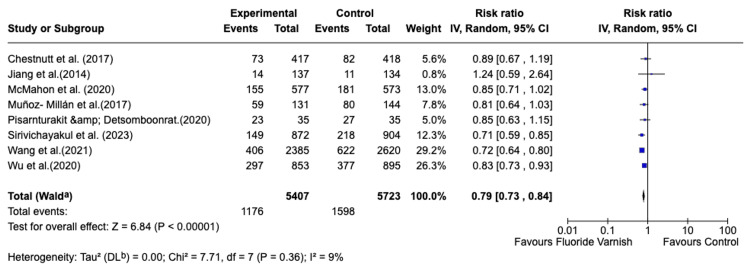
Forest plot of the meta-analysis comparing fluoride varnish versus control for caries prevention. Effect size is expressed as a risk ratio (RR) with 95%CIs, pooled using a random-effects inverse-variance model (DerSimonian–Laird τ²). The overall effect favors fluoride varnish (RR = 0.79, 95%CI 0.73–0.84; Z = 6.84, P < 0.00001). RR meta-analysis was performed using the random-effects model. Heterogeneity: χ² = 7.71, df = 7 (P = 0.36); I² = 9% (low heterogeneity detected). “Favours Fluoride Varnish” is positioned to the left of 1.0. ^a^CIs were calculated with the Wald method. ^b^Tau^2 ^calculated by DerSimonian–Laird method Studies included: [15,19,21-26]

Review Manager (RevMan) Version 9.10.0, 2024 (Cochrane, London, United Kingdom) was used to generate the forest plot of individual study RRs and the pooled meta-analysis result using the inverse variance random-effects model. The random-effects model was employed a priori to account for expected heterogeneity in FV formulations (concentration, compound type), application protocols (frequency, duration), population characteristics (age, baseline caries risk, fluoride exposure), and outcome measurement methods (WHO criteria, ICDAS, modified indices). All eight studies are positioned to the left of this line, with point estimates ranging from 0.71 to 1.24, indicating that all studies showed effects favoring FV (RR < 1.0), except that of Jiang et al. [19], which had a null estimate within confidence limits. The pooled risk ratio (RR = 0.79) represented by the diamond at the bottom, with a 95% CI (0.73-0.85), which does not cross the null value and lies well to the left of RR = 1.0. Figure 3 shows the forest plot of the meta-analysis. The pooled effect was driven primarily by Wang et al. (29.2%) [25] and Wu et al. (26.3%) [26], reflecting their large sample sizes; the remaining studies each contributed between 0.8% and 13.0% [15,16,19,21-23].

*Publication Bias Assessment: Funnel Plot Analysis* 

**Figure 4:**
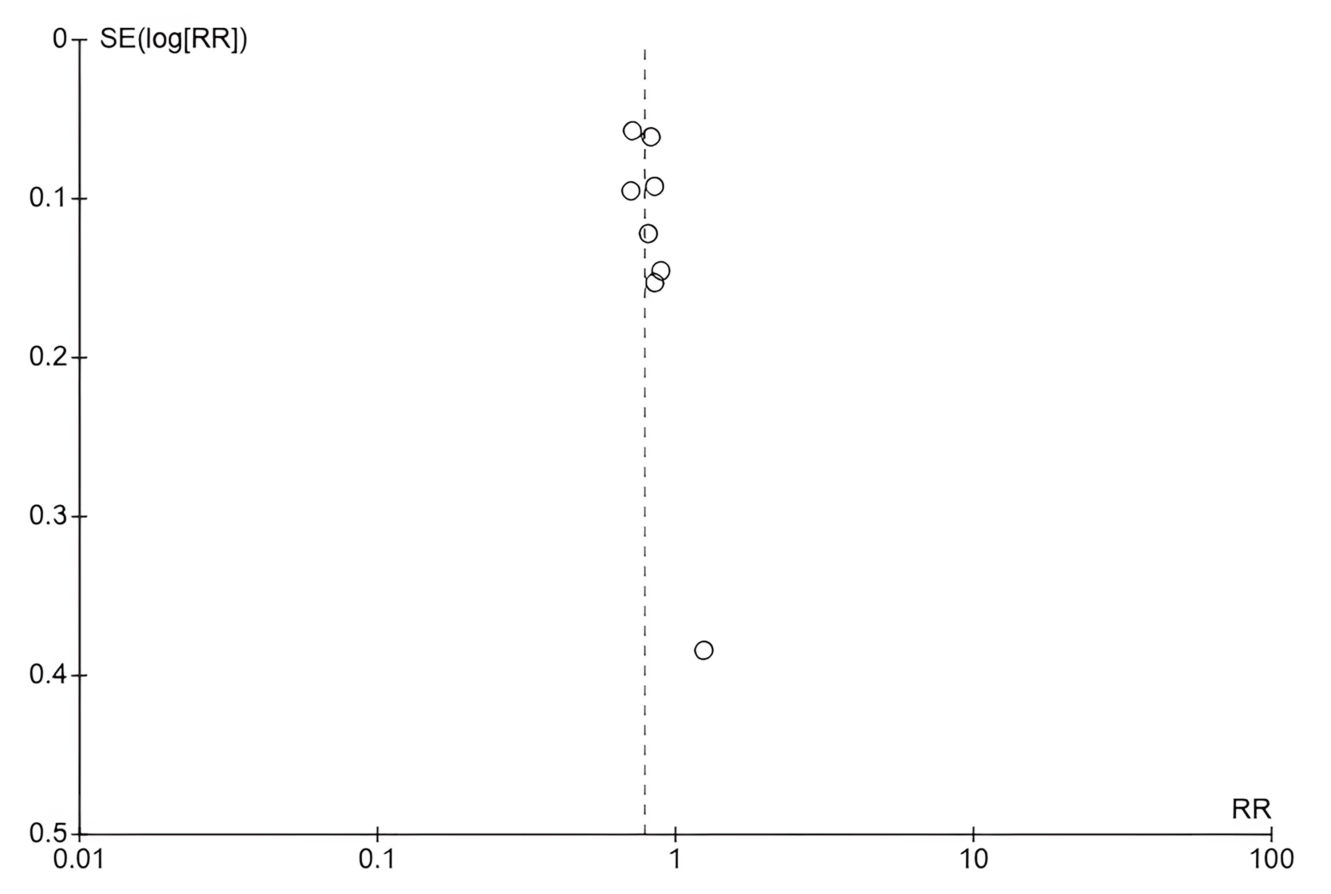
Funnel plot showing distribution of study effect sizes (risk ratio) against standard error. Each circle represents an individual study included in the meta-analysis. The horizontal axis shows the effect size (risk ratio (RR) on a logarithmic scale), while the vertical axis represents the study precision (standard error of the log RR). The vertical dashed line at RR = 0.79 indicates the pooled effect estimate derived from the random-effects model. The symmetric distribution of studies around the pooled effect line suggests the absence of significant publication bias. Combined with low heterogeneity (χ² = 7.71, df = 7, P = 0.36; I² = 9%), this indicates that the pooled results are robust and unlikely to be influenced by selective reporting. The studies (in order) from top left going downwards: Wang et. al (2022) [25], Wu et al. (2020) [26], McMahon et al. (2020) [21], Sirivichayakul et al. (2023) [24], Muñoz-Millán et al. (2017) [22], Chestnutt et al. (2017) [15], Pisarnturakit & Detsomboonrat (2020) [23], Jiang et al. (2014) [19]

To evaluate the potential influence of publication bias on the pooled results, a comprehensive funnel plot analysis was conducted following the standard Cochrane methodology and the recommendations of the GRADE framework. Figure 4 presents the funnel plot, where each circle represents an individual study. The horizontal axis depicts the effect size (risk ratio on a logarithmic scale), and the vertical axis represents study precision (standard error of the log risk ratio). By design, studies with higher precision (larger sample sizes) are positioned lower on the plot, while studies with lower precision (smaller sample sizes) appear higher. The vertical dashed line at RR = 0.79 represents the pooled effect estimate.

The funnel plot displays a symmetric distribution of the eight studies around the central effect line, indicating that publication bias is unlikely to have substantially influenced the overall findings. The absence of asymmetry suggests that the pooled estimate (RR = 0.79) accurately reflects the true treatment effect rather than an inflated or biased result. Combined with low heterogeneity (χ² = 7.71, df = 7, P = 0.36; I² = 9%), these results provide strong reassurance that the conclusions of this meta-analysis are robust and reflect the true state of the evidence on the effectiveness of FV.

Overall Certainty of Evidence (GRADE)

The overall certainty of evidence and pooled outcomes for FV compared with control were summarized according to the GRADE approach and are presented in Table 6.

**Table 6:** Summary of findings and certainty assessment for the effect of fluoride varnish compared with control. The table presents the pooled outcomes from eight randomized controlled trials (n = 11,130 participants). Risk ratios (RRs) were calculated using a random-effects model. The overall certainty of evidence was rated as high (⨁⨁⨁⨁) according to the GRADE criteria, with no serious concerns identified for risk of bias, inconsistency, indirectness, imprecision, or publication bias. The pooled analysis showed a 21.7% event rate in the fluoride varnish group versus 27.9% in the control group (RR = 0.79; 95% CI, 0.73–0.84), corresponding to 59 fewer events per 1,000 participants (range: 75 fewer to 45 fewer). CI, confidence interval; RR, risk ratio; GRADE: Grading of Recommendations Assessment, Development and Evaluation

Participants (studies)	Follow-up	Risk of bias	Inconsistency	Indirectness	Imprecision	Publication bias	Overall certainty of evidence	Study event rates (%)	Relative effect (95% CI)	Anticipated absolute effects	With Control	With Fluoride Varnish	Risk with Control	Risk difference with Fluoride Varnish
11130 (8 RCTs)		not serious	not serious	not serious	not serious	All plausible residual confounding would reduce the demonstrated effect dose response gradient	⨁⨁⨁⨁ High	1598/5723 (27.9%)	RR 0.79 (0.73 to 0.84)		1598/5723 (27.9%)	1176/5407 (21.7%)	1598/5723 (27.9%)	59 fewer per 1000 (from 75 fewer to 45 fewer)

Discussion

This systematic review derived data from 13,263 children across multiple continents and various socioeconomic and cultural environments. Its findings serve as a foundation for clinical and public health policies for dental caries prevention through school-based fluoride delivery programs. The central finding of this review is the efficacy of FVs in providing caries-preventive benefits across a spectrum of fluoride-delivery models. FVs maintain a significant preventive value in high-risk or under-fluoridated individuals; the observed preventative fractions range from 13% [16] to as high as 72% [13], depending on the study design, caries baseline, varnish concentration, and application frequency. The 2025 community-based systematic review by Griffin et al. corroborates these findings; they reported that SFVDP reduced caries initiation by 32% in permanent teeth and 25% in primary teeth [9]. The studies also have diverse baseline caries levels, from ultra-high-risk populations in South Africa (mean d1-4mft 5.9 ± 4.3) [17] to low-risk populations in Hong Kong (mean dmft score 0.03±0.24) [19].

The methodological aspects of the studies ranged from simple FV application to complex multi-component interventions. Muñoz-Millán et al. used FV as a standalone treatment but only achieved limited effectiveness (caries preventive fraction 18.9%) [22]. Whereas, McMahon et al. integrated FV into comprehensive Childsmile programs, including supervised brushing, and achieved modest additional benefits (26.9% had worsened d3mft (decayed, missing, or filled teeth, with decay reaching the dentin layer) in the FV group) [21]. Similarly, Jiang et al. compared parental education against combined parental education plus varnish, finding no additional benefit (11.9% vs. 17.5% ECC incidence) [19]. Wu et al. integrated oral health education with biannual varnish application, achieving significant effectiveness (58.9% vs. 65.5% caries prevalence in experimental vs control groups) [26]. On the contrary, Effenberger et al. combined FV with daily supervised tooth brushing but found no effectiveness (p > 0.05) [17]. The evidence suggests that by analyzing effectiveness across different intervention complexities and by identifying essential program components and eliminating ineffective add-on interventions, it is possible to optimize both clinical outcomes and resource utilization.

In this review, a key determinant of the success of school-based fluoride delivery programs emerged: age stratification. Jiang et al. [19] examined the youngest cohort (8-23 months), Agarwal et al. [13] focused on early childhood (3-4 years), Lam et al. [20] targeted preschoolers (3-4 years), Jayasinghe et al. [18] examined school-age children (6-7 years), Chestnutt et al. [15] analyzed the mixed dentition period (6-7 years) during permanent molar eruption, and Wu et al. [26] extended through late childhood (primary school age). Multiple studies targeting preschool children demonstrated the greatest effects (sometimes exceeding 70%) in the reduction of new carious lesions [13,19-23]. The Consensus Workshop on Child FV Program Implementation in Australia acknowledged the importance of the wider adoption of FV programs as part of oral health promotion efforts to prevent dental disease [11]. Studies in China [25] and Wales [15] demonstrate that as children get older, the focus of interventions rightly moves to the first permanent molars, especially during early school years. This suggests that clinical outcomes can be maximized at the population level when limited resources are used optimally by strategically matching preventive therapies with dental developmental milestones. 

A significant observation in the landmark study by Chestnutt et al. was that there were no significant differences in caries prevention between FV and fissure sealant applications for first permanent molars after 36 months of follow-up (OR = 0.86; 95%CI, 0.60 to 1.22) [15]. Similar findings were reported by Lam et al., who found that glass ionomer sealants, despite lower retention rates, had similar effects on caries prevention as sodium FV (p = 0.913) [20]. This challenges the fundamental principle of preventive treatments, that less technique-sensitive and easily administered interventions (such as FVs) can offer equivalent results to complex clinical interventions such as sealants. Thus, it is safe to conclude that intervention selection in real-world settings for large-scale programs should focus on scalability, feasibility, and effectiveness, rather than technically superior but resource-intensive restorative procedures.

Cost-Effectiveness and Sustainability

School-based fluoride programs provide significant economic benefits by improving oral health at lower costs per child than traditional clinical approaches. Four key studies assessed cost-effectiveness and sustainability alongside efficacy. Chestnutt et al. [15] and Effenberger et al. [17] concluded that FV was not cost-effective and raised sustainability concerns in deprived populations. McMahon et al. reported that FV was economically unfavorable (mean cost per child in the FV group was GBP 32.66; total cost was GBP 685.86 to prevent one child from having a worsening of d3mft) and stated caregiver participation and logistical issues as key sustainability factors [21]. On the contrary, Wu et al. inferred that FV was easy to apply and economical for caries prevention (it costs $22.23 to prevent caries in a first permanent molar. Whereas, a filling to restore a carious tooth costs $21.06, and a root canal treatment followed by a full-crown restoration costs $184.20); therefore, the implementation of FV application as a public health measure in schools in rural areas was recommended [26]. These findings were in agreement with those reported by Neidell et al. [27] and Amilani et al. [28] in their assessment of the cost-effectiveness of varnish in a school-based setting. Overall, these studies suggest that contextual and operational factors critically influence cost-effectiveness. 

Limitations and Shortcomings

Despite the compelling evidence, several methodological and practical limitations must be acknowledged. While the scope of the literature search was restricted (PubMed Central/MEDLINE, CENTRAL, Google Scholar), the high methodological quality of included studies (57.14% low risk of bias) and consistency of findings (I² = 9%) suggest that the evidence base adequately represents the current state of knowledge on school-based FV programs. Nonetheless, future comprehensive searches should include additional databases to ensure complete evidence capture. One major concern was the high rates of attrition. Four of the 14 studies reported notable attrition, decreased attendance, or dropout rates upwards of 30%, affecting statistical accuracy [13,22,23,26]. Second, the heterogeneity of the assessed criteria posed a challenge in data synthesis; the studies differed vastly in terms of target teeth/dentition (primary dentition, mixed dentition, first permanent molars, caries assessment on individual tooth surfaces, approximal caries, etc.), age ranges, baseline caries risk, setting variations, frequency of FV application, adjunctive therapies such as silver diamine FV or fissure sealants, and additional fluoride exposure (in the form of fluoridated water, toothpaste, etc.). Third, the absence of standardized outcome measures, especially regarding caries assessment, time points for outcome assessment, assessor/participant blinding, inconsistent reporting of outcome measures, and statistical presentation, also introduces variability in interpretation. Additionally, most studies had short- to medium-term follow-up durations, ranging from six to 36 months, with limited data beyond three years. Furthermore, few studies explicitly reported the use of intention-to-treat analyses, which limits the extent to which attrition bias could be mitigated. Finally, this review protocol was not prospectively registered, which may increase the potential for reporting bias, and this should be considered when interpreting the findings.

Research Priorities and Future Directions

This systematic review provides valuable evidence for the short- to medium-term effectiveness of FV in dental caries prevention. However, significant limitations remain in understanding the long-term outcomes and optimal implementation measures. Longitudinal cohort studies tracking participants into adulthood would help quantify the benefits of early dental intervention throughout an individual’s lifetime. In addition, mixed methods and implementation science approaches could provide valuable insights into program sustainability and fidelity in diverse real-world contexts. Thus, they would provide crucial information to support oral health policy development. 

Priorities for future research should include thorough economic evaluations of various program designs and comparative effectiveness studies of various implementation models. A thorough assessment of methods for preserving program fidelity and quality over long periods would also be beneficial. The current evidence base provides insights into the effectiveness of such programs in addressing oral health disparities. When implemented with attention to quality assurance, regional adaptation, implementation techniques, and inter-sectoral collaboration, these programs can achieve substantial community oral health improvements.

## Conclusions

School-based fluoride delivery programs are most effectively utilized as practical public-health initiatives rather than isolated clinical acts. When organized in schools, these programs help minimize avoidable gaps in oral health by reaching children who are less likely to receive preventive care elsewhere. In addition to their clinical benefits, these programs are highly adaptable across different health systems, with operational advantages like standardized workflows, predictable reach, and opportunities for quality assurance. Outcomes may differ between urban and rural settings, underscoring the importance of regional adaptation when designing and implementing such programs.

For future implementation, recommendations include matching the intensity and timing of application to developmental stages, integrating programs into everyday school routines, monitoring fidelity and outcome measures over time, and applying cost-aware designs that are realistic for services to sustain. At the same time, the short follow-up durations in most included studies should be considered when interpreting long-term effectiveness to avoid over-extrapolation. Overall, the evidence in this review supports school-based fluoride delivery as an effective method to achieve sustainable, population-level improvement in children’s oral health.
